# Joint metabolome and transcriptome analysis of the effects of exogenous GA_3_ on endogenous hormones in sweet cherry and mining of potential regulatory genes

**DOI:** 10.3389/fpls.2022.1041068

**Published:** 2022-10-18

**Authors:** Chaoqun Chen, Hongxu Chen, Yuanfei Chen, Wenlong Yang, Mengyao Li, Bo Sun, Haiyan Song, Wenjing Tang, Yao Zhang, Ronggao Gong

**Affiliations:** College of Horticulture, Sichuan Agricultural University, Chengdu, China

**Keywords:** sweet cherry, transcriptome, metabolome, endogenous hormones, wrky, gibberellin

## Abstract

Gibberellin (GA) is an important phytohormone that can participate in various developmental processes of plants. The study found that application of GA_3_ can induce parthenocarpy fruit and improve fruit set. However, the use of GA_3_ affects endogenous hormones in fruits, thereby affecting fruit quality. This study mainly investigates the effect of exogenous GA_3_ on endogenous hormones in sweet cherries. The anabolic pathways of each hormone were analyzed by metabolome and transcriptome to identify key metabolites and genes that affect endogenous hormones in response to exogenous GA_3_ application. Results showed that exogenous GA_3_ led to a significant increase in the content of abscisic acid (ABA) and GA and affected jasmonic acid (JA) and auxin (IAA). At the same time, the key structural genes affecting the synthesis of various hormones were preliminarily determined. Combined with transcription factor family analysis, *WRKY* genes were found to be more sensitive to the use of exogenous GA_3_, especially the genes belonging to Group III (*PaWRKY16*, *PaWRKY21*, *PaWRKY38*, *PaWRKY52*, and *PaWRKY53*). These transcription factors can combine with the promoters of *NCED*, *YUCCA*, and other genes to regulate the content of endogenous hormones. These findings lay the foundation for the preliminary determination of the mechanism of GA_3_’s effect on endogenous hormones in sweet cherry and the biological function of *WRKY* transcription factors.

## 1 Introduction

Sweet cherries (*Prunus avium* L.), belonging to the Rosaceae family, originated in Europe and Western Asia (Papapetros et al., 2018). Sweet cherries are rich in nutrients, such as flavonoids, ascorbic acid, and anthocyanins. These substances have beneficial effects on health, including preventive and regulatory effects on several chronic diseases (diabetes, cancer, cardiovascular, and other inflammatory diseases) (Faienza et al., 2020). Versatile and economical, sweet cherries are among the most popular fruits and are widely cultivated worldwide. However, sweet cherriy is a typical self-incompatible species controlled by the multi-allele expressed by a single gametophyte (Kivistik et al., 2022), and artificial pollination is often used to improve the fruit set. The workload of manual pollination is large and consumes considerable manpower and material resources. Therefore, people gradually adjust the program for higher efficiency, such as exogenous spraying of GA_3_ (Askarieh et al., 2021).

GA, an important plant hormone and signaling molecule, plays an important role in fruit reproductive development and stress response (Rachappanavar et al., 2022). Numerous studies have shown that GA affects fruit development and ripening, especially fruit-enhancing fruit sets. In pepper, exogenous use of GA_3_ can significantly increase yield (Tiwari et al., 2012). Treatment with GA_3_ during flowering stimulates cell division and ovary growth, thereby improving citrus fruit set, whereas paclobutrazol (GA_3_ biosynthesis inhibitor) inhibits cell division and reduces fruit set (Mesejo et al., 2016). Similarly, the use of GA_3_ before apple flowering can induce parthenocarpic fruit and increase the fruit setting rate (Watanabe et al., 2008). The accumulation of sucrose and organic acids in single-core pears induced by GA_4+7_ was lower than that in pollinated pears (Niu et al., 2015). Citrus carotenoids were significantly reduced after GA_3_ treatment (Zhang et al., 2012). Similarly, in sweet cherries, changes in fruit bioactivity and soluble sugars were found after the use of GA_3_ (Ozkan et al., 2016). Exogenous use of GA_3_ can improve the fruit set of sweet cherries, but the fruit quality changes (Kuhn et al., 2020).

Studies have shown that changes in endogenous hormones are one of the main reasons for the changes in fruit quality after GA_3_ is applied (Tijero et al., 2019). However, plant hormones do not play an independent role in the process of plant growth and development, but they interact with each other to form a complex multihormone regulatory network to jointly regulate the life activities of plants (Jaillais and Chory, 2010). Therefore, the relationship between hormones should be considered when studying the effect of exogenous GA_3_ on endogenous hormones. Studies have shown that GA_3_ and IAA induce cell wall expansion by activating the expression of *EXP* genes, the combination of which regulates stem formation (Kou et al., 2021). Meanwhile, GA and IAA regulated soybean lower ovule elongation under the interaction of low light and high-temperature stress (Bawa et al., 2020). Through the crosstalk between the ABA and GA signaling pathways, root growth and tillering can be maintained, and the plant structure can be regulated (Lin et al., 2020). GA, ABA, and IAA interact to regulate strawberry fruit development (Liao et al., 2018). ABA, JA, and SA together with ETH modulate some abiotic stress defense responses of trees exposed to sunlight during photooxidative and thermal stress (Torres et al., 2017). These findings further indicate a crosstalk mechanism between endogenous hormones to jointly regulate fruit growth, development, and quality.

At present, most studies on the effect of exogenous GA_3_ on the endogenous hormones in sweet cherry fruit focus on content determination, whereas systematic studies on the anabolic pathways of these hormones are limited. Therefore, in this study, the change patterns of endogenous hormones (ABA, GA_3_, GA4, IAA, and JA) were determined by spraying different concentrations of GA_3_. At the same time, by combining metabolome and transcriptome, the anabolic pathways of these hormones were analyzed to identify the differential metabolites (DEM) and differential genes (DEG) that affect endogenous hormones in response to exogenous GA_3_. In addition to this, we performed a comprehensive genome-wide analysis of the sweet cherry *WRKY* gene family. Gene identification, phylogenetic analysis, and analyses of gene structure, conserved motifs, promoter *cis-*elements, and protein-protein interactions were performed, respectively, to investigate the potential relevance of *WRKY* genes to the application of exogenous GA_3_. The results of this study will provide a reference for subsequent studies on the application of exogenous GA_3_ in sweet cherry production and lay a foundation for studying the biological function of sweet cherry *WRKY* transcription factors.

## 2 Materials and methods

### 2.1 Plant materials and processing

In this study, ‘Hongdeng’ sweet cherry was selected as the experimental material, and the experimental site was located in the sweet cherry experimental base of Hanyuan County, Ya’an City, Sichuan Province, China. A total of 24 sweet cherry fruit trees with good growing conditions and the same developmental period were selected for listing. One control (CK) and seven GA_3_ treatment concentration gradients were set up in the experiment: 10, 20, 30, 40, 60, 80, and 100 mg/L (Named A, B, C, D, E, F, G, respectively.), with three trees in each treatment. The entire tree was sprayed with GA_3_ meticulously at 9:00 a.m. in the early flowering period and one week after full bloom, and the control group was treated with clean water. Each sweet cherry fruit tree uses 5 liters of GA_3_ solution or water each time. Sampling was started after flowering, and then every three days until the fruit matured. A total of 12 periods of samples were collected (1, 2, 3, 4, 5, 6, 7, 8, 9, 10, 11, and 12). After harvesting, the fruits were brought back to the laboratory immediately, and then frozen in liquid nitrogen and stored in a −80°C refrigerator for subsequent experiments. Three biological replicates were prepared for each sample.

### 2.2 Hormone extraction and purification

Endogenous GA_3_, GA_4_, IAA, ABA and JA levels were determined using an indirect ELISA method. A 0.5 g sample was homogenized in liquid nitrogen and extracted in cold 80% (v/v) methanol containing 1 mM 2-tert-butylated hydroxytoluene as an antioxidant. The extracts were incubated at 4°C for 1 hour and centrifuged at 3500r/min for 8 minutes at the same temperature. The supernatant was then filtered through a Chromoseq C18 column (C18 Sep-Pak Cartridge, Waters, Millford, Massachusetts, USA). The resulting eluate was concentrated to dryness in vacuo and dissolved in 1 mL of phosphate buffered saline (PBS) containing 0.1% (v/v) Tween-20 and 0.1% (w/v) gelatin (pH 7.5) for ELISA analyze.

### 2.3 Metabolite profiling and data analysis

The freeze-dried sample was pulverized to a powder, and 100 mg was extracted with 600 μL of 2-chlorophenylalanine (4 ppm) in methanol overnight at 4°C. The supernatant was then collected by centrifugation at 12,000 rpm for 10 minutes. For each experimental sample, an equal volume of samples was obtained and mixed as a quality control (QC) sample, which was inserted in the front, middle, and back of the sample to test the repeatability of the experiment. Subsequently, these extracts were absorbed, filtered, and analyzed by a UHPLC-MS/MS system. ACQUITY UPLC^®^ HSS T3 1.8 μm (2.1 × 150 mm) columns were used in this study. Mobile phase A is positive and negative ion 0.1% formic acid-water solution, and mobile phase B is formic acid acetonitrile. Chromatographic gradient elution program: 0–1 min, 98% A, 2% B; 1–9 min, 74% A, 26% B; 9–12 min, 26% A, 74% B; 12–13.5 min, 2% A, 98% B; 13.5–14 min, 50% A, 50% B; 14–20 min, 98% A, 2% B. Raw data were processed using Compound Discoverer 3.1 (CD3.1). Functional and taxonomic annotations were performed on the metabolites to investigate the functional properties and taxonomy of the identified metabolites. The data were logarithmically transformed and centrally formatted using MetaX software (http://metax.genomics.cn/). Differential metabolites were screened by three parameters, variable importance in the projection (VIP), fold change (FC), and P-value. The thresholds were set as VIP > 1.0, FC > 1.2 or FC < 0.833, and P value < 0.05. Six independent replications were included for each sample. Finally, OmicShare tools (https://www.omicshare.com/tools/) were used to perform cluster heatmap, correlation, and metabolic pathway analysis of screened metabolites.

### 2.4 RNA extraction, library construction, RNA sequencing, and data analysis

Total RNA was extracted with a total RNA kit (TIANGEN Biotech, Beijing, China). Using the polyA structure at the end of mRNA, the sample mRNA was separated from the total RNA by Oligo (dT) magnetic beads, and the obtained mRNA was randomly interrupted with divalent cations in NEB Fragmentation Buffer reagent. Using the fragmented mRNA as a template, the first strand of cDNA was synthesized with random oligonucleotide primers. Then, the second strand of cDNA was synthesized by using dNTPs as raw material and DNA polymerase I, and the double-stranded cDNA fragment was purified and recovered. The purified double-stranded cDNA is end-repaired and A is added to the end, and then the sequencing adapter is ligated to the double-stranded cDNA. Fragment selection was performed on the size of the cDNA using AMPure XP beads, and the 200 bp sequence was enriched. The enriched sequences were amplified by PCR, the PCR products were purified using AMPure XP beads, and the library was further constructed. Qubit2.0 Fluorometer was used for preliminary quantification, and Agilent 2100 Bioanalyzer was used for quality inspection of the constructed library. After pooling, as required, Illumina sequencing (TSINGKE, Beijing, China) was performed to generate 150 bp paired-end reads. Gene expression levels were analyzed by the fragments per kilobase per million reads (FPKM) method. DESeq2 v1.22.1 was used for differential expression analysis between sample groups, the original readcount was normalized, the significant P-value was corrected using the Benjamini and Hochberg methods, and finally, the corrected P-value (p-adjust), which is the false discovery rate value (FDR). |log2 (Fold Change)| > 2 and p-adjust ≤ 0.05 were used as the screening criteria for the significance of differentially expressed genes. Kyoto Encyclopedia of Genes and Genomes (KEGG) analysis was performed using the clusterProfiler R package to clarify the signaling pathways involved in differential genes. The PlantTFDB database (http://planttfdb.gao-lab.org) was used to screen and classify possible transcription factors. At the same time, the log2(FPKM) values ​​of differential genes were used to draw a clustering heatmap. The clustering heatmap passed the TBtools software (http://www.tbtools.org) for drawing.

### 2.5 Retrieval and identification of members of the sweet cherry *WRKY* transcription factor family

The *WRKY* family module sequence (PF03106) was downloaded from the Pfam database (http://pfam.xfam.org/), and then the sweet cherry protein sequence was downloaded from NCBI (https://www.ncbi.nlm.nih.gov). The HMMER software was used to retrieve WRKY protein sequences from the sweet cherry genome sequence. Candidate proteins were further submitted to NCBI-CDD and Pfam for WRKY domain confirmation. The ExPASy website (http://web.expasy.org/protparam/) was used to analyze the physicochemical properties of the confirmed WRKY protein sequence, such as protein molecular weight and isoelectric point, and WOLF PSORT (http://www.genscript.com/wolf -psort.html) was use for subcellular localization analysis. The *WRKY* gene sequence of *Arabidopsisthalian* was downloaded from the TAIR (https://www.arabidopsis.org/) website.

### 2.6 Phylogenetic analysis of *WRKY* family in different species

Clustal X (v.2.1) software was used to perform multiple sequence alignment of all *WRKY* protein sequences of sweet cherry and *Arabidopsis thaliana*, and MEGA 6.06 software was used to perform phylogenetic analysis on the results of the multiple sequence alignment using the neighbor-joining method. The parameter bootstrap repeated 1000 times was verified, and a phylogenetic tree was constructed. The phylogenetic tree was modified in Evolview (http://www.evolgenius.info/evolview/#/login).

### 2.7 Sequence structure and conserved motif analysis of *WRKY* family members in sweet cherry

The GSDS server (http://gsds.cbi.pku.edu.cn/) was accessed, and the structural pattern diagram of the introns and exons of the *WRKY* family genes was designed by comparing the coding sequence with the gene sequence information. The conserved motifs of the members of the sweet cherry *WRKY* family gene were analyzed using MEME (http://meme-suite.org/). The parameter size of the conserved motif is set to be 10-100 amino acids, and the maximum number of domains to be exported is 10. Finally, the visualization of the results is realized by using TBtools software. The analysis results of these exon–intron structures and conserved motifs were arranged in the order shown on the phylogenetic tree.

### 2.8 Analysis of *cis-*acting elements of sweet cherry *WRKY* gene family

The Plant CARE (http://bioinformatics.psb.ugent.be/webtools/plantcare/html/) online software was used, considering the 2000 bp upstream of the start codon of the sweet cherry *PaWRKY* gene family as the sequence to predict and analyze the *cis-*acting elements of the gene family. Finally, the TBtools software was used to map.

### 2.9 Prediction of protein–protein interaction networks

All PaWRKY protein sequences were submitted to the STRING (https://cn.string-db.org) website, and *Arabidopsis thaliana* was selected as the reference organism. After blast analysis, the highest scoring *Arabidopsis* homolog (Bitscore) was used to construct the network. Genes that do not interact with any other genes are removed.

### 2.10 Correlation analysis of metabolite and transcript profiles

All the obtained DEMs and DEGs were mapped to the KEGG pathway database to obtain their common pathway enrichment information. The top 10 significantly enriched metabolic pathways in the three comparison groups were histogram plotted. The obtained DEM and DEG were analyzed based on the Pearson correlation coefficient, and the Cytoscape v3.9.1 software was used to make a correlation network diagram.

### 2.11 qRT-PCR analysis

Total RNA from the four samples was extracted with an RNA extraction kit (TSINGKE, Beijing, China). Subsequently, cDNA was synthesized using the Goldenstar RT6 cDNA Synthesis Kit Ver.2 kit (Beijing TsingKe Biotech Co., Ltd.). qRT-PCR was performed using the CFX96TM real-time system (Bio-Rad, California, USA) and 2 × TSINGKE^®^ Master qPCR Mix(SYBR Green I)(TSINGKE, Beijing, China) reagents. The amplification program was as follows: pre-denaturation at 95°C for 30 s, denaturation at 95°C for 0.05 s, annealing at 59°C for 30 s, and the number of amplification cycles was 39. Gene expression was normalized with ACTIN as an internal control. Gene expression was calculated using 2−ΔΔCt, and primers were designed using Primer Premier 6.0 software. **Table S1** lists the primers for qRT-PCR.

## 3 Results

### 3.1 Changes in endogenous hormones after exogenous GA_3_ treatment

**Figure 1:**
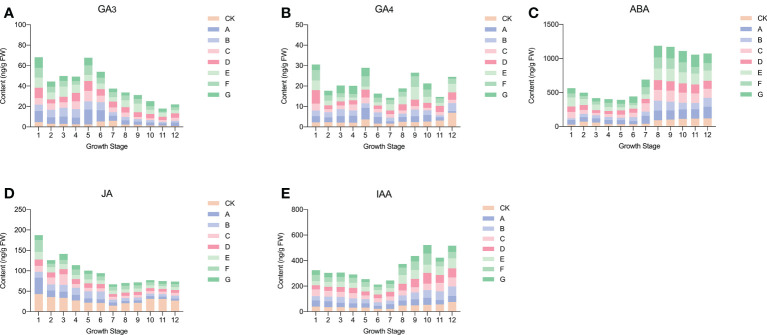
Changes of endogenous hormones in fruits after exogenous GA_3_ treatment. **(A)** Changes in endogenous GA_3_ after exogenous GA_3_ treatment. **(B)** Changes in endogenous GA_4_ after exogenous GA_3_ treatment. **(C)** Changes in endogenous ABA after exogenous GA_3_ treatment. **(D)** Changes in endogenous JA after exogenous GA_3_ treatment. **(E)** Changes in endogenous IAA after exogenous GA_3_ treatment.

The endogenous hormones in sweet cherry fruit changed after the use of exogenous GA_3_. The specific situation is shown in **Figure 1**. Among them, the GA_3_ content mainly showed a trend of initially decreasing and then increasing. The content of endogenous GA_3_ was significantly increased after exogenous GA_3_ treatment, and the change was most severe in the 5h to 6th period. In addition, the peak of GA_3_ in CK appeared in the 6th to 7th period, whereas the peak in each treatment group was advanced to the 4th to 6th period. GA_4_ generally showed an upward trend and reached its peak at maturity. After GA_3_ treatment, the content of endogenous GA_4_ was reduced in all periods except 1st, 6th, and 7th. ABA content, one of the most abundant hormones in sweet cherries, increased rapidly from stage 6, indicating that it may play a role mainly in the later stages of fruit growth. After treatment, the endogenous ABA content increased significantly, and the content increased the most in the 8th to 9th period, among which the E treatment group showed the greatest change, and the ABA content increased by approximately 80 ng/g FW. JA showed a trend of initially decreasing sharply to the 6th period and then increasing slightly. The lowest JA content in the seventh period was 20.71 ng/g FW. After GA_3_ treatment, the content of endogenous JA was down-regulated, and a slight difference was observed between the treatment groups. The overall IAA showed a trend of initially decreasing and then increasing, and reached a peak value at the mature stage, which was 74.15 ng/g FW. After treatment, except for E treatment, the other treatments reduced the content of endogenous IAA, and the effect was most severe in the 6th to 10th period.

### 3.2 Metabolome analysis

These results showed that after exogenous use of GA_3_, the effects of endogenous hormones were more severe in the three periods of the sixth, ninth, and 12th, and treatment group E had the most significant effect on endogenous GA_3_, ABA, and IAA in the three periods. Therefore, CK6, CK9, CK12, E6, E9, and E12 were selected for metabolome and transcriptome analysis.

**Figure 2:**
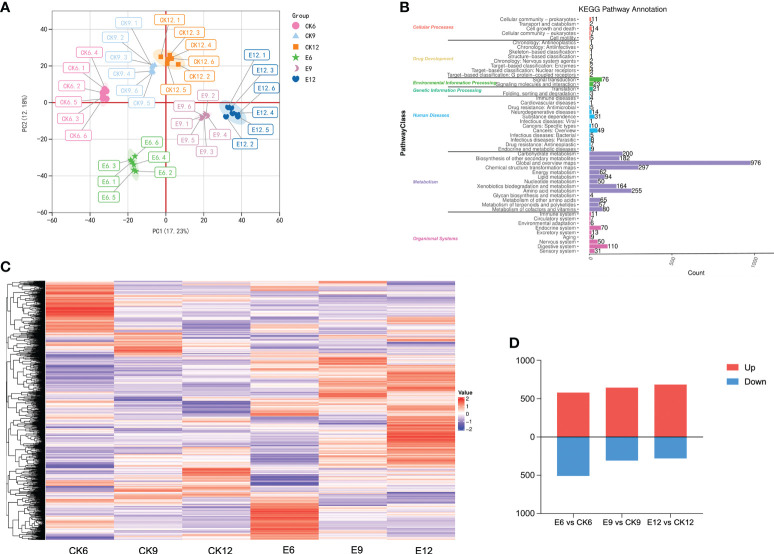
Metabolome analysis of fruits in three periods after gibberellin treatment. **(A)** PCA analysis of metabolites in different treatment groups. **(B)** KEGG Pathway classification annotation of metabolites. **(C)** DEMs expression heat map, color indicates the relative level content of each DEM, from low (purple) to high (red). **(D)** The number of DEMs in the treatment group and CK in the three periods, the number of up-regulated and down-regulated DEMs are represented by the bars above and below the x-axis, respectively.

The PCA results of the metabolome profiles are shown in **Figure 2A**, the first two principal components could separate 36 samples, accounting for 17.23% and 12.18% of the total variability. On the PCA analysis chart, each group showed a separation trend, and each replicate was clustered, indicating that the data reproducibility was good during the experiment. Evident differences were observed among the six sweet cherry samples; CK6, E6, and CK9 were distributed in the positive end of PC1, whereas CK12, E9, and E12 were distributed in the negative end of PC1. In addition, in PC2, the CK group was distributed on the positive end, and the processing group E was distributed on the negative end. A total of 3011 metabolites were identified in the metabolome, including 2013 positive and 998 negative ions (**Figure 2B**). Through KEGG functional annotation, the identified metabolites were divided into seven categories, of which the metabolism group had the most metabolites, reaching 2486, accounting for 79.81%.

Thresholds were set to VIP > 1.0, FC > 1.2 or FC < 0.833, and P-value < 0.05 to screen for DEM. A total of 2256 DEMs were identified, and their expression patterns are shown in **Figure 2C**. Significant differences were found among the groups, and the higher expression of many DEMs in the treatment groups may be the main substance causing the differences in endogenous hormones in fruits. Each CK was compared with group E at different times, as shown in **Figure 2D**. Among the combinations of different treatments, the combination with the most differential metabolites was E6 vs CK6 (1088 in total, 580 up-regulated, and 508 down-regulated). In summary, fruit metabolites were changed in multiple periods after the exogenous use of GA_3_ changed, and most expression levels showed an upward-regulated trend.

### 3.3 Transcriptome analysis

**Figure 3:**
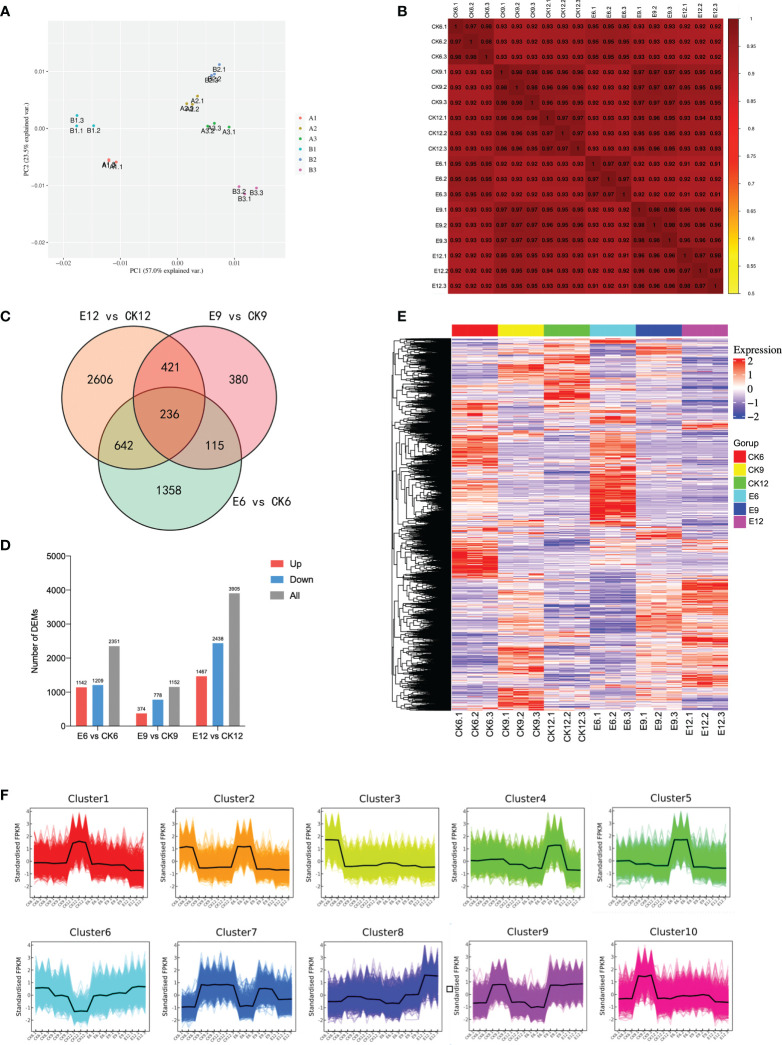
Transcriptome analysis of fruits in three periods after gibberellin treatment. **(A)** PCA analysis of gene expression under different treatments. **(B)** Spearman correlation coefficient of gene expression under different treatments. **(C)** Venn plot of commonly and exclusively expressed DEGs in the comparison of control and treatment groups at each developmental stage. **(D)** The number of DEGs compared at any two different developmental stages, the number of up-regulated and down-regulated genes is represented by the bars above and below the X-axis, respectively. **(E)** Hierarchical clustering of DEGs of all samples. **(F)** K-means clustering of DEGs expression trends, the expression profiles of genes in each cluster are represented by different colors, and the average expression profiles of all genes in each sample are represented by a black line.

The samples of each treatment group were analyzed by RNA-seq technology. A total of 441,209,814 raw data were generated in three periods. After filtering out linker sequences, uncertain reads, and low-quality reads, 798,325,520 high-quality clean reads were obtained, and an average of 93.25% of the clean reads were mapped to the sweet cherry genome (Detailed results are shown in **Table S2**). The expression of transcript samples was analyzed by PCA, and the results are shown in **Figure 3A**. The figure shows that each sample can be clearly distinguished on the score map, and the resets are closely focused, indicating that the fruit transcripts are different after using exogenous GA_3_. Similar to metabolome, CK6 and E6 are at the minus end of PC1, and the remainder is at the plus end of PC2. Interestingly, the score map shows that the discrimination between CK12 and E9 on PC1 is weak, indicating that the two samples are similar. Moreover, the sample gene expression correlation between the replicates of each sample was the highest, indicating that the samples had good repeatability, and CK12 and E9 had a high degree of correlation (**Figure 3B**).

Differential gene screening was performed with a threshold of P-value < 0.05 and |log2FoldChange|>2, and a total of 10,154 DEGs were identified. The results of DEGs compared between different developmental stages are shown in **Figure 3C**. Among the three groups, E12 vs CK12 had the most DEGs, reaching 3905. Different from the metabolome results, E12 vs CK12 had more differential genes than E9 vs CK9, and the number of up-regulated DEGs was less than that of down-regulated DEGs. The results of analyzing common or unique DEGs between the three comparison groups are shown in **Figure 3D**. The genes expressed in each sample were the least, only 236, and the genes expressed only in E12 vs CK12 were the greatest (2606).

Furthermore, a hierarchical clustering heatmap was drawn for the three developmental periods, with good repeatability of each treatment and large transcriptional differences between groups (**Figure 3E**). The expression patterns of all DEGs were divided into 10 groups, with more highly expressed genes in CK6, E6, and E12 (**Figure 3F**). Cluster2 contained the most DEGs, reaching 1593, mainly expressed in CK6 and E6. The expression levels of most DEGs were up-regulated in E12 vs CK12, mainly in Cluster8 and Cluster9.

### 3.4 Combined metabolome and transcriptome analysis

#### 3.4.1 KEGG enrichment analysis

**Figure 4:**
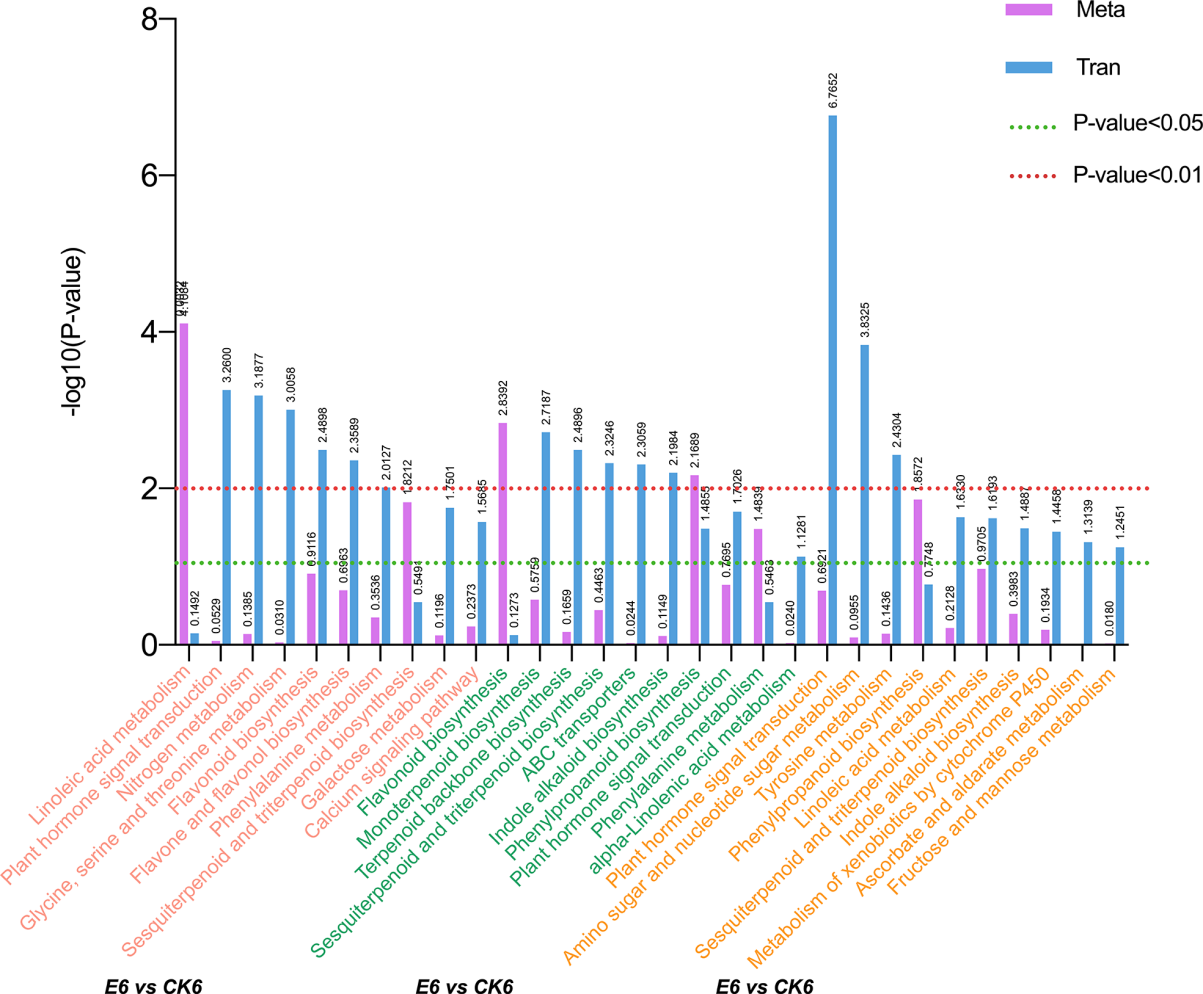
Annotation analysis of KEGG pathway in comparison groups of each period after gibberellin treatment. Select DEGs with P-value ≤ 0.5 for KEGG enrichment analysis.

A KEGG pathway analysis was performed to further determine the main biochemical pathways and signal transduction pathways jointly participated by DEGs and DEMs, and the results are shown in **Figure 4**. The figure shows the top 10 significantly enriched metabolic pathways for each comparison group, with a total of 21 different pathways. Among them, the pathways that were significantly enriched in the top 10 in the three comparison groups were sesquiterpenoid and triterpenoid biosynthesis and plant hormone signal transduction. In the comparison group of E12 vs CK12, plant hormone signal transduction had the smallest p-value and was the most significant pathway. Meanwhile, we found that pathways, such as flavonoid biosynthesis and phenylalanine metabolism, were significantly enriched in the E6 vs CK6 combination, suggesting the probable differences in fruit coloration after GA_3_ treatment. In addition, the enrichment of fructose and mannose metabolism pathways was higher in E12 vs CK12, indicating that the use of exogenous GA_S_ may affect the sugar content of sweet cherries.

#### 3.4.2 Analysis of hormone anabolic pathways

**Figure 5:**
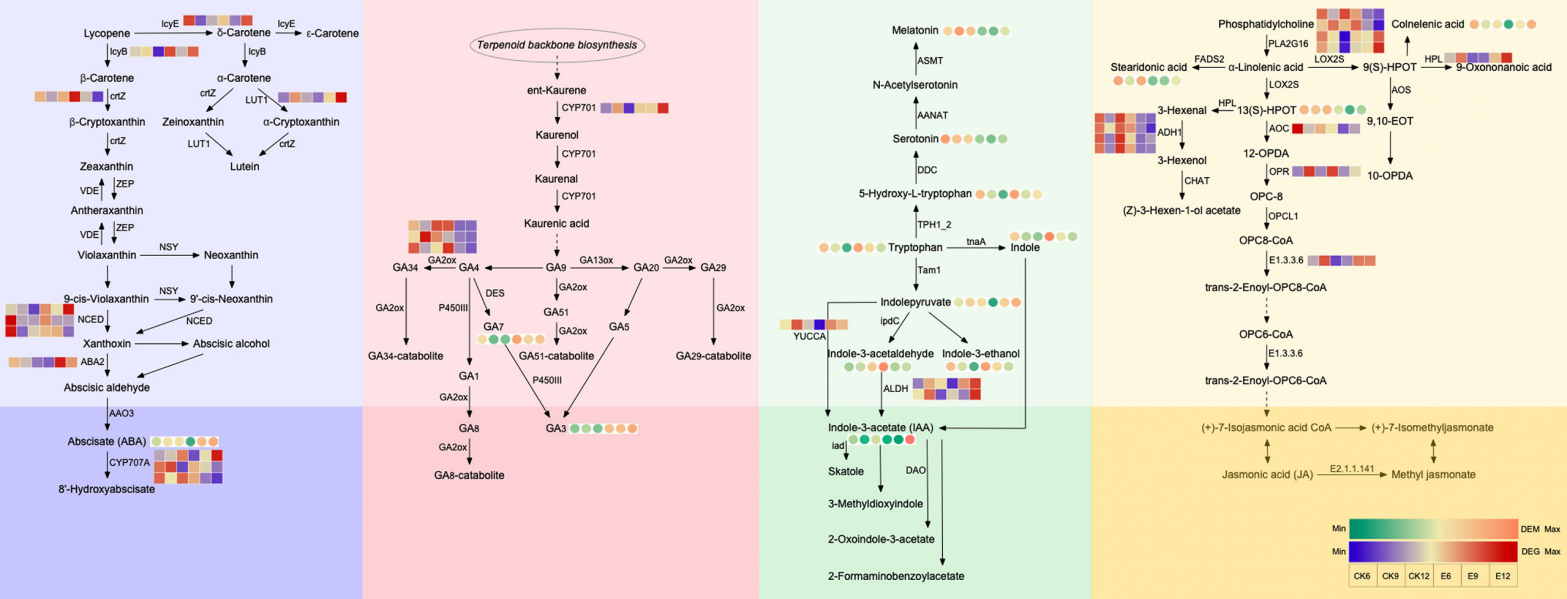
Analysis of each hormone anabolic pathway. The circle heatmap represents the expression of DEMs, and the square heatmap represents the expression of DEGs.

Therefore, we carried out a detailed analysis of the anabolism of the five hormones, and the results are shown in **Figure 5** and **Table S3**. In the ABA synthesis pathway, ABA is the only DEM, which gradually increases with fruit ripening. After the administration of exogenous GA_3_, the content of ABA was significantly changed, especially in the latter two periods. The ABA content of E9 was 3.79 times higher than that of CK9, whereas that of E12 was 7.42 times higher than that of CK12. The expression of two *NCED* genes and one *ABA2* gene increased after the use of GA_3_, which promoted the synthesis of xanthoxin and abscisic aldehyde and established sufficient precursor substances for the accumulation of ABA. At the same time, the expression of two *CYP707A* genes was inhibited after treatment, resulting in the massive accumulation of ABA. Interestingly, we found that the expressions of *lcyE* and *LUT1* genes were significantly up-regulated in E9 and E12 after GA_3_ treatment, indicating that the use of exogenous GA_3_ may affect the content of δ-Carotene and Lutein in fruits.

The metabolic pathway of GA indicates that exogenous GA_3_ has a greater impact on the content of endogenous GA_3_ than GA_4_. Similar to the previous results (**Figure 1A**), exogenous GA_3_ significantly increased the content of endogenous GA_3_ in the three periods. At maturity, the expression of endogenous GA_3_ in the treatment group increased by 39717583.24 compared with CK. At the same time, we found that the content of GA_7_ changed significantly, thereby providing a sufficient material basis for the increase in GA_3_. In this pathway, the expression of *CYP701* and *GA2ox* genes was significantly up-regulated and down-regulated, respectively, after treatment, promoting the massive synthesis of GA_7_ and GA_3_.

The DEMs in the IAA synthesis pathway are more abundant. The use of exogenous GA_3_ decreased the content of Melatonin and Serotonin, but increased the contents of 5-Hydroxy-L-tryptophan, tryptophan, and indole, especially in E6. The accumulation of these substances lays a sufficient foundation for the synthesis of IAA. At the same time, the *ALDH* gene regulating IAA synthesis was highly expressed after treatment, especially the expression of E12 increased by 2.61 times compared with CK12. *YUCCA* gene expression was also up-regulated.

DEMs in the JA synthesis pathway are mainly concentrated in the anterior part. Exogenous GA_3_ decreased the contents of 13(S)-HPOT and stearidonic acid, and the expression of *AOC* and *OPR* genes was inhibited after treatment. Therefore, the repressed expression of these genes may be the main reason for the reduction of endogenous JA content.

In summary, the expression of *NCED*, *ABA2*, *CYP701*, *ALDH*, and other genes was affected after GA_3_ treatment; thus, the contents of endogenous ABA, GA_3_, and IAA changed.

### 3.5 Transcription factor family analysis

**Figure 6:**
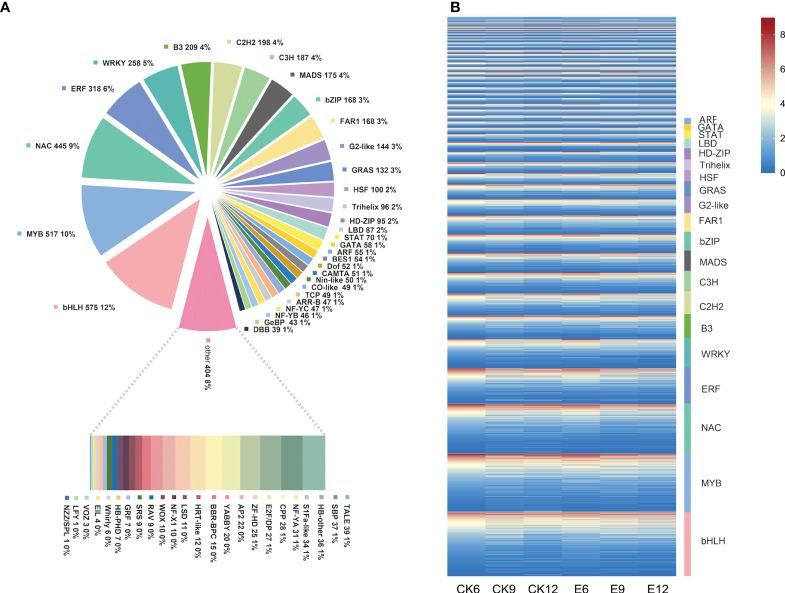
Transcription factor analysis. **(A)** Statistical summary of transcription factor families, the number and proportion of each transcription factor family are displayed in a pie chart. **(B)** Heat map of transcription factor expression patterns. The FPKM values of transcription factors were transformed according to log2, and heatmaps were constructed using Tbtools software.

In the transcription factor family analysis, 4986 DEGs were identified as transcription factors, belonging to 55 transcription factor families. The transcription factor family mainly includes bHLH, MYB, NAC, and WRKY. The specific transcription factor family is shown in **Figure 6A**. Among them, transcription factor families, such as bHLH and MYB, accounted for the highest proportion, that is, 11.53% and 11.52%, respectively. Moreover, the proportion of transcription factor families, such as NAC (8.92%), ERF (6.38%), WRKY (5.17%), and B3 (4.19%) was relatively high.

Therefore, the expression patterns of these transcription factors were further analyzed, as shown in **Figure 6B**. Most transcription factors were expressed in each treatment, and their expression was affected by exogenous GA_3_. Among them, the expression of transcription factors, such as MYB and bZIP, showed a downward trend after treatment. However, transcription factors, such as MYB and bHLH, were closely related to the synthesis of anthocyanin in fruit, indicating that the use of exogenous GA_3_ may affect fruit color. Interestingly, the expression of most WRKY transcription factors was increased after exogenous GA_3_ treatment, and more genes were up-regulated in E6.

#### 3.5.1 Identification of sweet cherry *PaWRKYs*

The results obtained show that the expression of genes related to the synthesis of hormones is up-regulated after exogenous use of GA_3_, thereby promoting the accumulation of these endogenous hormones. Therefore, according to the expression patterns of these genes, further screening was performed in cluster4, cluster5, cluster6, cluster8, and cluster9 to further identify the transcription factors that regulate these endogenous hormone synthesis genes. Among them, the genes of the *WRKY* family had the largest number of differential genes among the five groups, indicating that *WRKY* may be the main internal regulator in response to changes in exogenous GA_3_.

According to the HMMER search results, we finally obtained 58 sequences with typical *WRKY* domains, namely, *PaWRKY1*–*PaWRKY58*, using the Pfam tool to identify the domains. The physicochemical properties (gene name, gene ID, amino acid size, molecular weight, theoretical isoelectric point, and subcellular localization) were analyzed according to the sequence, and the results are shown in **Table 1**. The results showed that the amino acid size, molecular weight, and isoelectric point of these *PaWRKY* genes exhibited great differences. The protein encoded by the *PaWRKY26* gene has the shortest amino acid length, containing only 97 amino acids, and its protein molecular mass is 11708.2 Da. The longest amino acid is PaWRKY8 protein, which contains 740 amino acids, its protein molecular mass is 79829.63 Da, and its theoretical isoelectric point is predicted to be 4.92–9.92. According to the subcellular localization analysis, most genes were mainly located in the nucleus, similar to the WRKY transcription factor genes reported in other species.

**Table 1:** Basic information of members of *PaWRKYs* gene family.

Gene name	Gene ID	Amino acids	Molecular weight/Da	Theoretical pI	Group	WoLF PSORT
*PaWRKY1*	LOC110767577	437	47414.97	4.92	II-e	nucl
*PaWRKY2*	LOC110749399	280	32165.39	5.08	II-d	nucl
*PaWRKY3*	LOC110762470	314	35398.78	5.11	II-e	nucl
*PaWRKY4*	LOC110751810	357	39763.26	5.15	III	nucl
*PaWRKY5*	LOC110764860	558	61645.64	5.2	II-b	nucl
*PaWRKY6*	LOC110760656	356	39964.36	5.24	III	nucl
*PaWRKY7*	LOC110769738	269	29407.61	5.25	II-e	nucl
*PaWRKY8*	LOC110765346	162	18707.37	5.34	II-c	nucl
*PaWRKY9*	LOC110771999	334	37718.47	5.47	III	nucl
*PaWRKY10*	LOC110768952	323	36275.34	5.47	III	nucl
*PaWRKY11*	LOC110756877	506	55912.98	5.49	I	nucl
*PaWRKY12*	LOC110767143	350	38306.45	5.61	III	nucl
*PaWRKY13*	LOC110771993	339	38129.3	5.64	III	nucl
*PaWRKY14*	LOC110767511	740	79829.63	5.7	I	nucl
*PaWRKY15*	LOC110771978	357	40735.01	5.74	III	nucl
*PaWRKY16*	LOC110756098	282	30821.59	5.8	II-e	nucl
*PaWRKY17*	LOC110768951	371	40804.43	5.81	III	nucl
*PaWRKY18*	LOC110751361	330	36066.52	5.81	II-c	nucl
*PaWRKY19*	LOC110749956	486	52935.3	5.84	I	nucl
*PaWRKY20*	LOC110752494	389	42694.75	5.89	II-c	nucl
*PaWRKY21*	LOC110760420	738	80782.4	5.93	I	nucl
*PaWRKY22*	LOC110752839	546	59639.64	5.95	II-b	nucl
*PaWRKY23*	LOC110764924	523	56802.51	6.04	II-e	nucl
*PaWRKY24*	LOC110758282	587	63999.36	6.07	I	nucl
*PaWRKY25*	LOC110764283	650	70731.02	6.16	II-b	nucl
*PaWRKY26*	LOC110771979	336	37449.64	6.21	III	nucl
*PaWRKY27*	LOC110751302	633	69129.29	6.38	II-b	nucl
*PaWRKY28*	LOC110752464	506	55452.73	6.52	II-b	nucl
*PaWRKY29*	LOC110745365	196	22119.27	6.52	II-c	nucl
*PaWRKY30*	LOC110745366	196	22119.27	6.52	II-c	nucl
*PaWRKY31*	LOC110763060	612	66386.49	6.66	II-b	nucl
*PaWRKY32*	LOC110766559	334	37034.8	6.68	II-c	nucl
*PaWRKY33*	LOC110755676	321	35641.8	6.75	II-c	nucl
*PaWRKY34*	LOC110753889	590	64696.37	6.77	I	nucl
*PaWRKY35*	LOC110767257	536	59527.31	6.82	I	nucl
*PaWRKY36*	LOC110754876	364	41145.25	6.87	II-c	nucl
*PaWRKY37*	LOC110751328	516	56174.74	7.28	I	nucl
*PaWRKY38*	LOC110750648	244	27715.36	7.29	II-c	nucl
*PaWRKY39*	LOC110750649	244	27715.36	7.29	II-c	nucl
*PaWRKY40*	LOC110752057	683	73473.62	7.61	II-b	nucl
*PaWRKY41*	LOC110762547	327	36551.11	7.62	II-a	nucl
*PaWRKY42*	LOC110755743	490	52847.36	7.68	II-b	nucl
*PaWRKY43*	LOC110751738	357	39319.46	7.68	II-e	nucl
*PaWRKY44*	LOC110755553	530	58044.2	7.74	I	nucl
*PaWRKY45*	LOC110762653	285	31618.43	8.54	II-a	nucl
*PaWRKY46*	LOC110763398	320	35314.43	8.77	II-a	nucl
*PaWRKY47*	LOC110767234	475	51742.18	8.91	I	nucl
*PaWRKY48*	LOC110764429	223	25562.39	8.97	II-c	nucl
*PaWRKY49*	LOC110772429	239	27237.52	9.03	II-c	nucl
*PaWRKY50*	LOC110756733	210	24000.97	9.08	II-c	pero
*PaWRKY51*	LOC110751840	221	24558.69	9.24	II-c	nucl
*PaWRKY52*	LOC110760359	170	19398.71	9.37	II-c	nucl
*PaWRKY53*	LOC110748572	97	11708.2	9.41	II-c	nucl
*PaWRKY54*	LOC110774544	342	37313.06	9.47	II-d	nucl
*PaWRKY55*	LOC110769338	184	20809.31	9.56	II-c	nucl
*PaWRKY56*	LOC110763432	355	40071.31	9.59	II-d	nucl
*PaWRKY57*	LOC110746394	326	35616.29	9.6	II-d	nucl
*PaWRKY58*	LOC110758321	286	31070.13	9.92	II-d	nucl

nucl means nucleus; pero means peroxisome.

#### 3.5.2 Analysis of phylogenetic relationship, gene structure, conserved motifs, and conserved elements in promoter regions

**Figure 7:**
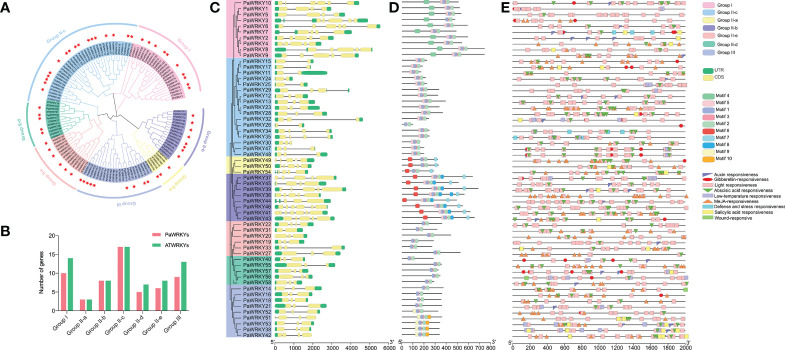
*WRKY* gene analysis of sweet cherry. **(A)** The phylogenetic relationship of the WRKY gene in sweet cherry. After aligning the sequences with Clustal W, a phylogenetic tree was constructed according to the neighbor-joining method. **(B)** The number statistics of *PaWRKYs* and *AtWRKYs* in each subfamily. **(C)** WRKY of sweet cherry Gene families were constructed based on the alignment of full-length amino acid sequences to construct a neighbor-joining tree and analyze gene structure. **(D)** Conserved motif analysis of the *WRKY* gene family in sweet cherry. **(E)** Analysis of functional elements in the promoter region of the *WRKY* gene family in sweet cherry.

This study selected *Arabidopsis thaliana* and sweet cherry to construct a phylogenetic tree to analyze the evolutionary relationship of the *PaWRKY* gene family (**Figure 7A**). A total of 58 PaWRKY proteins and 70 ATWRKY proteins showed evident clustering in the phylogenetic tree. According to gene clustering, the *PaWRKY* gene family can be divided into three subfamilies, Group I, Group II, and Group III. The Group II subfamily can also be subdivided into five subfamilies: Group II-a, Group II-b, Group II-c, Group II-d, and Group II-e. Among the seven subgroups, Group II-a subgroup contains the least number of *WRKY* genes and only three in sweet cherry and *Arabidopsis*. The largest *WRKY* gene family is Group II-c, which contains 17 *PaWRKY* genes and 17 *ATWRKY* genes. The second groups containing more *WRKY* genes were Group I (10 *PaWRKYs* and 14 *ATWRKYs*) and Group III (9 *PaWRKYs* and 13 *ATWRKYs*). The number of *WRKY* transcription factor family members contained in each combination is shown in **Figure 7B**.

According to the gene structure analysis (**Figure 7C**), all 58 members of the *PaWRKY* gene family have coding regions (CDS) and untranslated regions (UTR), of which the longer intron sequence is *PaWRKY6*. At the same time, *PaWRKYs* with higher sequence similarity are similar in structure; for example, the coding regions of *PaWRKYs* belonging to Group III are similar in length and structure. Among them, the gene structure of *PaWRKYs* in Group III is more conserved than that of other subfamilies.

In addition, to better understand the conserved structure in the PaWRKY protein sequence, MEME software was used to analyze the PaWRKY protein structure, and 10 different conserved motifs were identified (**Figure 7D**). Most *PaWRKY* members located in the same subfamily have similar conserved motifs, which are consistent with the grouping of the family phylogenetic tree, showing a certain arrangement. All *PaWRKY* genes contain the most basic family motif 1, motif 2, and motif 3. In addition, gene members of the Group I subfamily contain motif 4, Group III specifically motif 10, Group II-b specifically motif 8, and Groups II-a and II-b specifically motifs 6 and 7, respectively. This finding suggests that the reason why *WRKY* family members of different subfamilies are involved in coordinating specific processes of fruit growth and development may be that they have specific conserved structures.

To elucidate the possible regulatory mechanism of the conserved elements in the promoter region of the *PaWRKY* genes, *cis*-element analysis was performed on the 2000 bp upstream of the *PaWRKYs* gene family in sweet cherry. Nine *cis*-acting elements related to hormones and abiotic stresses were screened among the numerous response elements (**Figure 7E**). In terms of the number of elements, the light-responsive elements are the most, and they are distributed in the promoter regions of each *PaWRKY* gene. Every gene contains at least one hormone action element. Among them, ABA-acting and MeJA-acting elements are more abundant than other hormones.

#### 3.5.3 Expression pattern analysis of *PaWRKYs*

**Figure 8:**
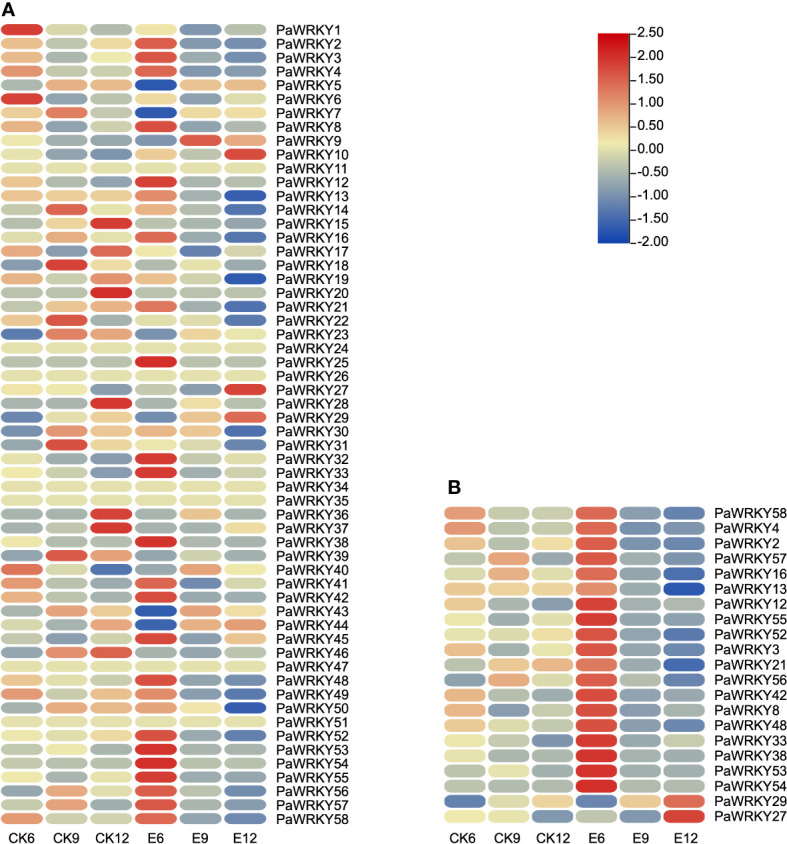
Expression profile of *PaWRKYs* genes. **(A)** The expression profile of *PaWRKYs* gene in three periods after treatment, and the FPKM values of transcription factors were transformed according to log2. **(B)** The relative expression levels of differentially expressed *PaWRKYs* family members after gibberellin treatment. The FPKM values of transcription factors were transformed and plotted according to log2.

The expression patterns of *PaWRKY* genes were analyzed according to the FPKM values determined by transcriptome analysis (**Figure 8A** and **Table S4**). Among them, except for *PaWRKY11*, *PaWRKY24*, *PaWRKY26*, *PaWRKY34*, *PaWRKY35*, *PaWRKY47*, and *PaWRKY51*, the expression of the remaining genes was detected in at least one group. The expression patterns of *PaWRKY* genes had high similarity in different subgroups. During sweet cherry development, the expression of most *PaWRKY* genes increased with fruit growth, such as *PaWRKY5*, *PaWRKY15*, and *PaWRKY37*. In CK, the expression of *PaWRKYs* was generally low, and after treatment, the expression of most genes was up-regulated.

The up-regulated expression of these genes may be positive regulators of endogenous hormone synthesis. The differentially expressed *PaWRKY* genes were screened in the transcriptome, and the results are shown in **Figure 8B**. The figure shows that exogenous use of GA_3_ generally up-regulated the expression level of the 6th stage and inhibited the expression of the 9th and 12th stages. On the contrary, we found that the high expression of *PaWRKY27* and *PaWRKY29* was at E12, consistent with the changing pattern of *NCED*, *CYP701*, and other genes.

#### 3.5.4 Protein interaction analysis of *PaWRKYs*

**Figure 9:**
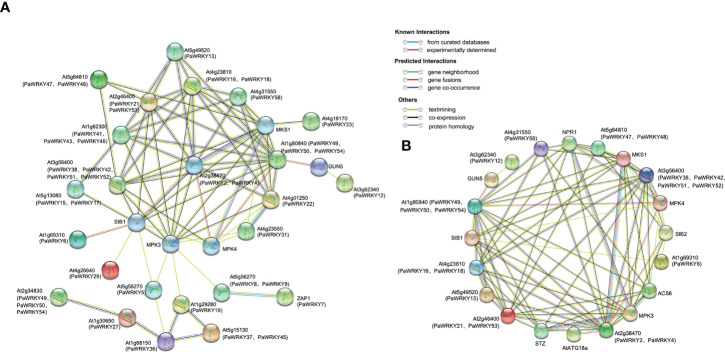
Protein interaction network. **(A)** Protein interaction network based on *Arabidopsis* homologous PaWRKYs. Abbreviated names are genes reported in *Arabidopsis*. The PaWRKYs proteins homologous to *Arabidopsis* are shown in parentheses. **(B)** PaWRKYs protein interaction network prediction based on differential expression of homology in *Arabidopsis*. Abbreviated names are genes reported in *Arabidopsis*. The PaWRKYs proteins homologous to *Arabidopsis* are shown in parentheses.

The cognate *WRKYs* of *Arabidopsis thaliana* were used to predict the protein interaction network of PaWRKYs (**Figure 9A**, see the **Tables S5** and **S6** for detailed annotation information in the figure). The results showed that most PaWRKY proteins interact with multiple proteins, of which 15 proteins can interact with more than four other PaWRKY proteins. For example, PaWRKY2 and PaWRKY4 are expected to interact directly with 13 WRKY proteins, namely, PaWRKY58 (AT4G31550.1 ortholog), PaWRKY18 (AT4G23810.1 ortholog), PaWRKY16 (AT4G23810.1 ortholog), PaWRKY21 (AT2G46400.1 ortholog), and others.

**Figure 9A** shows that differential genes have more complex relationships in protein interaction prediction. Therefore, we selected PaWRKYs with complex interactions in **Figure 9A** to further predict protein interaction networks (**Figure 9B**). These differential PaWRKYs (PaWRKY2, PaWRKY4, PaWRKY12, PaWRKY13, PaWRKY16, PaWRKY21, PaWRKY29, PaWRKY38, PaWRKY48, PaWRKY52, PaWRKY53, PaWRKY54, and PaWRKY58) can directly interact with various proteins, such as MKS1, SIB1, and ACS6. Among them, PaWRKY2 and PaWRKY4 (AT4G31550.1 ortholog) interact directly with 13 proteins, indicating that they may have important regulatory roles. In addition, PaWRKY21, PaWRKY16, PaWRKY53, and PaWRKY58 interact with stress-related proteins, such as MKS1 and MPK4, in response to growth and developmental changes. Overall, the predicted network provides an important reference for functional studies of PaWRKY proteins.

### 3.6 Correlation network analysis

**Figure 10:**
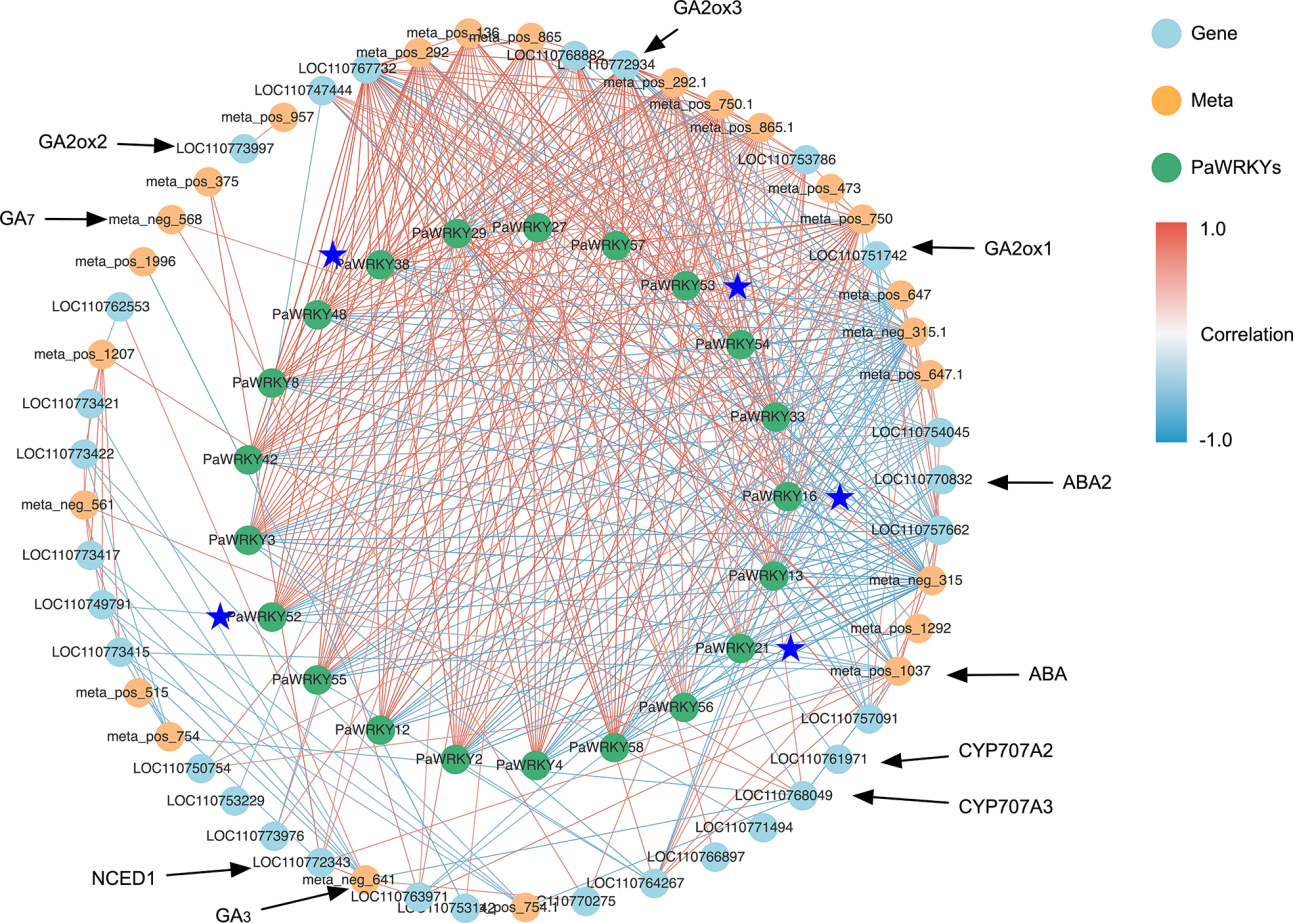
Correlation network analysis. The blue points represent genes, the yellow points represent metabolites, and the green points represent *PaWRKYs*; the red line represents positive correlation, the blue line represents negative correlation, and the darker the color, the higher the correlation; the genes marked with asterisk belong to *PaWRKYs* of Group III.

Based on these studies, the *PaWRKY* genes may play an important role in the growth of sweet cherries. Therefore, the correlation analysis of transcription factors, hormone synthesis-related genes, and related metabolites was performed using Cytoscape software, and the results are shown in **Figure 10**. The figure shows that these *PaWRKYs* are closely related to the structural genes involved in the synthesis of various hormones. Among them, *PaWRKY29* has a significant positive correlation with ABA and Indolepyruvate and has a strong correlation with *NCED*. It is a potential positive regulator of ABA and IAA synthesis. Similarly, *PaWRKY27* may have the same effect. In addition, *PaWRKY38* was positively correlated with *crtZ*, and the expression of *crtZ* was inhibited by exogenous GA_3_, suggesting that *PaWRKY38* may inhibit ABA synthesis. Moreover, *PaWRKY38* was significantly negatively correlated with *GA2ox2*, which inhibited the decomposition of GA_4_ and promoted the accumulation of GA_3_. Interestingly, the differential genes belonging to Group III (*PaWRKY16*, *PaWRKY21*, *PaWRKY38*, *PaWRKY52*, and *PaWRKY53*) were significantly negatively correlated with indolepyruvate, and their correlations ranged from −0.87 to −0.75. These results suggest that genes within Group III are more sensitive to the use of exogenous GA_3_.

### 3.7 qRT-PCR validation

**Figure 11:**
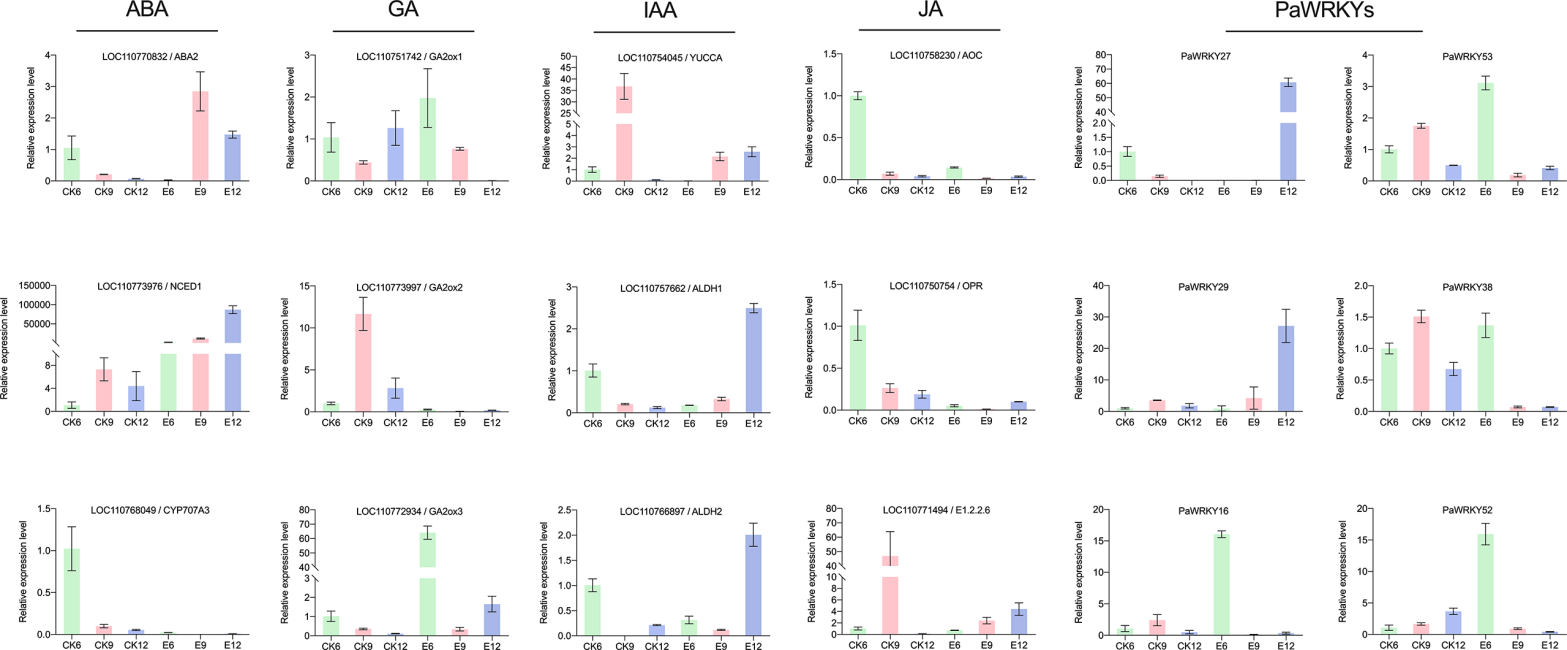
qRT-PCR analysis of genes and transcription factors related to hormone synthesis in sweet cherry after exogenous GA_3_ treatment.

Key genes sensitive to exogenous GA_3_ were selected from each pathway for qRT-PCR analysis to verify the validity of the transcripts, and the results are shown in **Figure 11**. The use of exogenous GA_3_ increased the expression of *NCED1* and *ABA2*, thereby increasing the synthesis of ABA. In addition, two *CYP707A* genes were inhibited, and the decomposition of ABA was inhibited. The significant decrease in the expression of *GA2ox* after treatment may be the main reason for the accumulation of GA_3_. The direct regulatory genes *YUCCA* and *ALDH* in the IAA synthesis pathway were highly expressed at E12, which in turn promoted the increase in IAA. JA synthesis-related genes were less sensitive to exogenous GA_3_, but were generally inhibited and showed low expression. At the same time, some *WRKY* genes of *PaWRKY27*, *PaWRKY29*, and Group III were selected for qRT-PCR verification. Overall, the qRT-PCR results for most structural genes and transcription factors were consistent with the transcriptome data, indicating a high level of confidence in the transcript data.

## 4 Discussion

Self-incompatibility is a serious problem in sweet cherry production, affecting fruit yield and restricting the development of the planting industry (Wu et al., 2013). Artificial pollination can solve this production problem, but the accompanying labor costs increase the cost of sweet cherry production. The study found that exogenous use of GA_3_ can improve fruit set, reduce labor costs, and effectively solve the cost problem of sweet cherry production (Devasirvatham and Tan, 2022). Aliyu et al. (2011) found that GA_3_ was the most effective phytohormone to improve fruit yield in cashew nuts treated with various phytohormones at the flowering stage. In addition, similar conclusions are found on sweet cherries. Exogenous use of GA_3_ can significantly promote parthenocarpic fruit and increase fruit yield (Wen et al., 2019). These results showed the importance of GA_3_ to the growth of sweet cherry fruit.

In plants, GA_3_ is a plant hormone with various biological functions. It can not only stimulate plant growth and development, but also mediate various stress responses (Hou et al., 2013). However, the fruit quality also changed after the exogenous use of GA_3_. When pineapples were treated with GA_3_ at the flowering stage, the higher concentration indicates higher volume of pulp cells, and the increase in fruit weight becomes more significant (Li et al., 2011). In citrus production, the method of spraying GA_3_ is often used to reduce the phenomenon of peeling and puffing of citrus, but it brings the problem of the delayed coloring of citrus (Ma et al., 2021). Liu et al. (2022) treated apples with exogenous GA to control cell growth at flowering and young fruit stages, thereby reducing asymmetric fruit. Chen et al. (2020) found that by regulating the level of GA_3_, the shape and ripening of tomato fruit can be affected. The study found that exogenous use of GA_3_ often affects fruit quality by affecting the content of endogenous hormones. For example, exogenous use of GA_3_ increased endogenous GA_3_ content in citrus and inhibited the rate of fruit browning (Cai et al., 2021). Liu et al. (2011) found that exogenous GA_3_ could regulate the growth of tiller buds by changing the endogenous ABA, IAA, and ZR contents of rice plants. Therefore, understanding the changing pattern of endogenous hormones after exogenous use of GA_3_ is greatly important to explore the mechanism of fruit quality changes after GA3 treatment. In this study, after exogenous use of GA_3_, the content of endogenous ABA, GA_3_, GA_4_, IAA, and JA changed with the concentration of use, and the hormone with the most drastic change was ABA. The contents of ABA and GA_3_ increased significantly after exogenous GA_3_ treatment, whereas the contents of GA_4_ and JA were inhibited. Only when the treatment concentration was 60 mg/L, did the content of IAA increase. These results indicated that the effect of exogenous GA_3_ on fruit yield and quality may be through changing the content of endogenous hormones. This is a systematic study on the changing pattern of endogenous hormones after administering exogenous GA_3_. This provides a theoretical reference for the application of GA_3_ in the production of sweet cherries and some ideas for further research on the effect of GA_3_ on the quality of stone fruit.

We further used the combined transcriptome and metabolome analysis to determine the relevant metabolites and genes in response to exogenous GA_3_ and identified a total of 2256 DEMs and 10154 DEGs. A large number of metabolites and genes jointly respond to the use of exogenous GA3 to regulate the content of endogenous hormones, thereby affecting the growth and development of sweet cherry fruit and changing fruit quality (Information on the differential metabolites related to the synthesis and metabolism of fruit sugars, acids and flavonoids is shown in **Table S7**. In this study, the expression levels of *NCED* and *ABA2* were significantly up-regulated, thereby directly affecting the accumulation of endogenous ABA. Similar findings were also found in grapes, where *NCED* and ABA3 affect fruit quality by participating in ABA biosynthesis (Li et al., 2021). At the same time, the study found that exogenous use of allantoin (Moriyama et al., 2020), acetic acid (Sun et al., 2022), and synthetic strigolactone analogue (Ferrero et al., 2018) improved fruit quality by affecting the expression of the *NCED* gene. The finding indicates that *NCED* is highly sensitive to external conditions and is the main regulator of ABA synthesis. In the GA pathway, the expression of the three *GA2ox* genes that regulate GA_4_ breakdown was significantly inhibited during maturation, and the expression of the *CYP701* gene was significantly upregulated after treatment, indicating that *GA2ox* and *CYP701* cooperate to promote the massive accumulation of GA_7_ and GA_3_. In the study of alkaline stress, the authors found that alkaline stress also changed the content of endogenous GA_3_ by changing the expression of *GA2ox* (Ma et al., 2022). Bermejo et al. (2018) also made similar conclusions in the study of strawberry, the expression of *GA2ox* was significantly changed after exogenous use of IAA. Similarly, exogenous use of GA_3_ and paclobutrazol modulates anthocyanin accumulation in Arabidopsis by affecting *GA2ox* (Zhang et al., 2017). In tomatoes, specific overexpression of SlGA2ox1 reduced endogenous GA concentrations in the fruit (Chen et al., 2016). In the study of IAA synthesis, we found that after exogenous use of GA_3_, a large number of DEMs appeared, whereas DEGs were less, indicating the importance of *YUCCA* and *ALDH* genes. Similar findings were also found in loquat (Jiang et al., 2016). After exogenous use of GA_3_, the expression of the *YUCCA* gene was up-regulated, increasing the IAA content. Compared with the four other hormones, the content of JA was significantly inhibited and down-regulated. The use of exogenous GA_3_ mainly acts on the genes in front of the JA synthesis pathway, such as *OPR*, *AOC*, and *E1.3.3.6*. In Arabidopsis, *AOC* and *OPR* are also key regulatory genes for JA synthesis (Leon-Reyes et al., 2010).

Transcription factor family molecules indicated that *WRKY* transcription factors were greatly affected by exogenous GA_3_, and the difference ratio was higher. *WRKY* transcription factors play crucial roles in regulating various plant growth and developmental processes. However, the *WRKY* gene family of the sweet cherry has not been widely studied, and its role remains to be explored. Therefore, we systematically analyzed the *WRKY* gene family of sweet cherry. The 58 *PaWRKY* gene families were studied in detail by analyzing the phylogeny, gene structure, promoter region, and sequence characteristics. However, the number of *WRKY* genes in sweet cherries was less than that in apple (127) (Meng et al., 2016), kiwifruit (97) (Jing and Liu, 2018), and pear (103) (Huang et al., 2015), which may be caused by the differences between plant genomes. We classified the 58 PaWRKY proteins into seven subfamilies on the basis of phylogeny. Similar findings were also found in the study of strawberry FaWRKY protein, and the 47 *FaWRKY* genes were divided into seven subfamilies (Chen and Liu, 2019). Gene structure and conserved motif analysis of *PaWRKYs* showed that genes belonging to the same subfamily had similar exon and intron organization and similar conserved motifs. These results suggest that *PaWRKYs* of the same subfamily are closely related in evolution. Among the seven subgroups, Group II contained the largest number of subgroups and genes, indicating its variability. According to previous research results, Group III gene members have the highest activity and play an important role in plant evolution (Wu et al., 2005). In this study, although Group III genes had fewer members, most genes showed differences after the use of GA_3_, suggesting that it may be a potential regulator of fruit growth. In addition, the protein function prediction indicated that *PaWRKY16*, *PaWRKY21*, and *PaWRKY53* could directly interact with multiple proteins to regulate fruit growth.

*WRKY* gene also plays an important role in regulating the content of plant endogenous hormones. Wang et al. (2016) isolated a new *WRKY* gene *CsWRKY2* from Camellia, which can participate in the signaling pathway of ABA synthesis, regulate ABA synthesis, and further improve plant defense against cold and drought stress. *CaWRKY40*, found in pepper, can be induced by JA-mediated signaling mechanisms, coordinating call responses to heat stress (Dang et al., 2013). In tobacco, overexpression of *NtWRKY50* resulted in altered JA levels and increased plant resistance (Liu et al., 2017). *WRKY* gene can cooperate with structural genes related to endogenous hormone synthesis to influence endogenous hormones. For example, Yan et al. (2014) found that the cotton transcription factor *GhWRKY17* can reduce ABA levels by inhibiting the expression of the *NCED* gene, thereby regulating the sensitivity to drought. Similarly, *GhWRKY1* was found to interact with the “W-box” *cis*-elements of the promoters of *AtNCED2*, *AtNCED5*, *AtNCED6*, and *AtNCED9* in Arabidopsis to promote ABA biosynthesis (Hu et al., 2021). In Pyrus betulaefolia, *PbrWRKY53* can bind to the W-box element in the promoter region of *PbrNCED1* to promote the synthesis of vitamin C and ABA, thereby improving drought tolerance (Liu et al., 2019). In addition, *WRKY* was found to be involved in the IAA signaling process in plants (Jin et al., 2018). The correlation between the *WRKY* gene and IAA metabolites or genes in this study also indicates that the *PaWRKY* gene in sweet cherries may be involved in the IAA signaling process

In summary, the *WRKY* gene can participate in the signal response process of various plant hormones, such as ABA and JA, and is widely involved in the growth and development of plants. In this study, potential regulatory genes in response to exogenous GA_3_ changes were initially screened by a combination of transcription and metabolism methods, which laid the foundation for sweet cherry fruit production. However, the specific regulatory mechanism of *WRKY* transcription factor and endogenous hormone synthesis related structural genes is still unclear and requires further analysis.

## Data availability statement

The datasets presented in this study can be found in online repositories and Supplementary Material. The metabolome and transcriptome proposed in the study are deposited in the National Genomics Data Center database. You can query the metabolome data by visiting the link (https://ngdc.cncb.ac.cn/omix/release/OMIX001762) (BioProject: PRJCA010046; Accession number: OMI001762); You can query transcriptome data by visiting the link (https://ngdc.cncb.ac.cn/gsa/browse/CRA007287) (BioProject: PRJCA010046; accession number: CRA007287).

## Author contributions

RG and CC: conceptualization. CC and HC: data curation. YC and WY: formal analysis. CC, WT, HS, and YZ: investigation. CC and HC: software. CC: writing–original draft. ML, BS, RG, and HS: writing–editing. RG: supervision. All authors read and approved the final manuscript.

## Funding

This research was funded by the Sichuan Science and Technology Plan Project (Key R&D Project) (2021YFN0081, 2021YFN0082). The funders had no rolein the design of the study in the collection, analyses, or interpretation of data, in the writing of the manuscript, or in thedecision to publish the results.

## Conflict of interest

The authors declare that the research was conducted in the absence of any commercial or financial relationships that could be construed as a potential conflict of interest.

## Publisher’s note

All claims expressed in this article are solely those of the authors and do not necessarily represent those of their affiliated organizations, or those of the publisher, the editors and the reviewers. Any product that may be evaluated in this article, or claim that may be made by its manufacturer, is not guaranteed or endorsed by the publisher.
